# *Streblus asper* attenuates alloxan-induced diabetes in rats and demonstrates antioxidant and cytotoxic effects

**DOI:** 10.1080/13880209.2021.1954668

**Published:** 2021-08-07

**Authors:** M. Oliur Rahman, Ali S. Alqahtani, Sayma Binte Huda, Shah Alam Siddiqui, Omar M. Noman, Fahd Nasr, Md. Abul Hassan, Sheikh Nazrul Islam

**Affiliations:** a Department of Botany, University of Dhaka, Dhaka, Bangladesh; b Department of Pharmacognosy, College of Pharmacy, King Saud University, Riyadh, Saudi Arabia; c Department of Applied Chemistry and Chemical Engineering, Islamic University, Kushtia, Bangladesh; d Institute of Nutrition and Food Science, University of Dhaka, Dhaka, Bangladesh

**Keywords:** Antidiabetic, DPPH, free radical scavenging, cytotoxicity, Moraceae

## Abstract

**Context:**

*Streblus asper* Lour. (Moraceae) is used for the treatment of different ailments, including diabetes, and requires scientific validation.

**Objective:**

The study evaluates antidiabetic effects, antioxidant potential, and cytotoxicity of leaf and bark extracts of *S. asper*.

**Materials and methods:**

Antidiabetic effects were assessed by inducing diabetes in Wistar albino rats (*n* = 5, six groups included 30 rats) by injecting alloxan [0.25 mg/kg body weight (bw)] intraperitoneally, and efficacy of methanol extracts of leaf and bark, and aqueous extract of leaves were evaluated by oral administration of 300 mg/kg bw of extracts for 3 weeks. Glibenclamide (Dibenol™) was used as a control (10 mg/kg bw). Antioxidant properties were examined by DPPH free radical scavenging activity, and cytotoxicity was investigated using a brine shrimp lethality assay.

**Results:**

Methanol extracts of leaves and bark, and the aqueous extract of leaves of *S. asper*, caused significant reductions in blood glucose levels in diabetic rats of 36.83, 70.33, and 52.71%, respectively, after 21 days of treatment. IC_50_ values in DPPH radical scavenging assessment for those extracts were 58.92, 88.54, and 111.36 µg/mL, respectively. LC_50_ values for brine shrimp lethality for the extracts were 173.80, 32.36, and 3235.9 µg/mL, respectively.

**Discussion and conclusions:**

The methanol bark extract of *S. asper* showed significant antidiabetic activity. This study will significantly contribute to establishing the plant as an alternative medicinal resource for rural populations of Bangladesh and provides an opportunity for further research to identify the primary active compound(s) and establish new drug candidates.

## Introduction

Lack of proper insulin functioning or insufficient insulin secretion results in diabetes mellitus, causing exaggerated deposition of glucose within the body’s systems (Bolen et al. 2007), which affects body functions in maintaining proper sugar levels in the bloodstream. Diabetes may restrict normal flow, damage blood vessels, and cause major complications, such as stroke or heart attack, nephropathy, retinopathy, and even neuropathy (Williams and Siraj 2012). The International Diabetes Foundation (IDF) reports that complications of diabetes are the third leading cause of mortality worldwide, which puts diabetes just behind high blood pressure and tobacco-initiated diseases. The percentage of the world population with diabetes is estimated to increase from 8.8 to 10.47% (642 million) by 2040 (IDF 2013).

Bangladesh is one of the countries having the highest rate of diabetes among the people (IDF 2013). As reported by IDF, the number of detected diabetes patients in Bangladesh is 7.1 million in a population of about 160 million, and an equal or even higher number may have undetected diabetes (IDF 2013). This high diabetes incidence imposes a negative influence on the country’s health care system. Most diabetes patients are living below the poverty level, have no access to modern medical treatment, and ultimately depend on local complementary/conventional medicines.

Various traditional preparations are administered for treating different complications resulted from diabetes worldwide (Nahas and Moher 2009). Ethnobotanical studies have explored a wide number of plant preparations that are used as a healing agent for treating diabetes complications and other ailments (Abo et al. 2008; Skalli et al. 2019). However, only a few are scientifically examined to evaluate claimed activity (Karan et al. 2013; Rahman et al. 2018; Atolani et al. 2019).

Oxidation is a normal physiological process, but can at times lead to the production of excessive oxygen radicals that may exceed normal antioxidant capacity and results in oxidative stress. When excessive oxygen radicals or reactive species are not neutralized through the internal cellular mechanism, such as catalyzing, peroxidation, and superoxide dismutation (SOD) by antioxidants or enzymes, oxidative damage to proteins, lipids, and DNA occurs. This damage gives rise to cytotoxicity, genotoxicity, and even carcinogenesis when damaged (mutated) cells proliferate (Ames et al. 1993). Several synthetic antioxidants have been widely used in the last few decades. Recently, many studies have reported that chronic use of some synthetic antioxidants is hazardous and may even lead to carcinogenesis (Sharma and Pandey 2009). Therefore, the discovery of safer natural antioxidants is currently urgently needed.

*Streblus asper* Lour. (Moraceae) is an ethnomedicinally important small tree, characterized by its straggling branchlets with short stiff hairs, elliptic-ovate to elliptic coriaceous leaves, puberulous fragrant perianth, white stamens, and globose ovary. This species is used in the Indian subcontinent ethnomedicinally for the treatment of different diseases, including leprosy, piles, diarrhoea, dysentery, and elephantiasis (Kumar et al. 2011). In Bangladesh, the *Marma* ethnic tribe uses the juice of roots of *S. asper* to treat irregular menstruation and promote delayed menstruation (Rastogi et al. 2006). A decoction of bark and seeds of *S. asper* is used for the treatment of diarrhoea and dysentery by the tribal healers of hilly districts of Bangladesh (Morshed and Nandni 2012), while aqueous extracted leaf juice is applied to treat diabetes (Uddin et al. 2019).

Different chemical classes of compounds have been isolated from roots of *S. asper*, e.g., cardiac glycosides, β-sitosterol, lupanol, and vijaloside (Rastogi et al. 2006). Recently, seven compounds viz. *trans*-streblusol A, *erythro-*streblusol B, *threo-*streblusol B, *R*-streblusol C, streblusquinone, streblusol D, and streblusol E have been isolated from the stem bark of *S. asper* (Li et al. 2012). The acid-base fractions of *S. asper* have been studied for antibacterial, antioxidant, anti-acetylcholinesterase, and neuroprotective activities (Prasansuklab et al. 2018). The species has also been investigated for its anti-cancer effects (Rawat et al. 2018; Zhang et al. 2021), anti-inflammatory activities (Singsai et al. 2020), and antidiarrheal effects (Shahed-Al-Mahmud et al. 2020). Previous studies depict that petroleum ether extract and ethanol extract of the stem bark (Karan et al. 2013), and methanol extract of root bark (Kumar et al. 2012) of *S. asper* have already been studied for their anti-diabetic effect. However, no scientific report is available to support the traditional use of this species, i.e., consumption of aqueous extract of stem bark and leaves of *S. asper* for the treatment of diabetes in Bangladesh. The methanol extract of stem bark and leaves have not been investigated as well so far. Therefore, in our present study, we assess the antidiabetic and antioxidant properties of aqueous extract and methanol extract of stem bark and leaves of *S. asper*. Despite ethnomedicinal uses of different preparations of *S. asper* are administered in Bangladesh, very little is known about its toxicity. Prolonged and safe use of the traditional herbal preparations does not always guarantee the preparation as safe; consequently, screening on its toxicity is required to overcome the short- or long-term toxic effect on human health. Therefore, in our study, we also primarily assess the cytotoxicity of the extracts of *S. asper* using Brine Shrimp lethality assay. This study will significantly contribute to establishing the plant as alternative medicinal resource in Bangladesh, where affording conventional modern medicine is farfetched for most of its inhabitants due to poverty.

## Materials and methods

### Plant sample

Leaves and bark of *S. asper* were collected on 20 October 2018 from the compound of the University of Dhaka. The plant was identified by Prof. Dr. Md. Abul Hassan and Prof. Dr. M. Oliur Rahman of the Department of Botany, University of Dhaka, Bangladesh, and the voucher specimen has been preserved at Dhaka University Salarkhan Herbarium (Accession no. DUSH 10,888). All plant parts were dried at room temperature before grinding into powder using a grinding mill. The powders were then passed through a mechanical sieve shaker to remove coarse materials above the size of 100 μm. This fine powder was used for organic solvent extractions.

### Chemicals

Alloxan monohydrate, glibenclamide (Dibenol™) obtained from the Square Pharmaceuticals Ltd. Bangladesh, methanol from Merck (Darmstadt, Germany), DPPH, BHT, DMSO (dimethyl sulphoxide), and vincristine sulphate (VS) manufactured by Sigma Aldrich (St. Louis, MO, USA). Other reagents were analytical grade.

### Preparation of plant extract

Powered leaf and bark, 150 g each, were soaked in 300 mL of distilled methanol for 4 days with occasional shaking and stirring. Samples were then filtered with a thin fine cloth and then filter paper. The filtrates were evaporated to dryness under reduced pressure at 40 °C using a rotary evaporator to give a 5.8 g (yield from dry powder, 3.86%) and 6.0 g (yield from dry power, 4.0%) of residue for leaves and bark, respectively.

For the preparation of aqueous leaf extract, fresh leaves (1000 g) were ground with distilled water and filtered with filter paper which yielded 9.6 g (yield from fresh leaves, 0.96%) of aqueous extract.

### Antidiabetic study

#### Collection and acclimatization of rats

Healthy adult Wistar albino rats, 90–120 g body weight (bw) of either sex, aged 4–5 weeks were collected and housed individually in cages at 24 °C with a relative humidity of 55–65% with a 12 h light/dark cycle and fed formulated rat food and water. Selected rats were kept for one week to adapt to laboratory conditions before experimentation. The guidelines of the National Institute of Health for the Care and Use of Laboratory Animals (NIH Publication revised in 1996) were followed in this study.

#### Preparation of alloxan solution and induction of diabetes

The weights and blood glucose levels were measured after fasting of animals for 12 h using ACCU-ANSER DIGITAL blood glucometer. Alloxan monohydrate was dissolved in normal saline and a freshly prepared solution was administrated orally to animals at a dose of 100 mg/kg bw. Animals were allowed free access to food and water 30 min after injection and after 72 h the blood glucose of the rats was measured again. Animals with glucose levels >10 mmol/L were selected for investigation.

#### Preparation of sample suspension and glibenclamide suspension

Calculated amounts of sample were taken for preparing suspensions of each type of plant extract of 300 mg/kg bw (Khatun et al. 2015). A suitable amount of distilled water was added to prepare every suspension. A small amount of Tween-20 and gum acacia was added to dissolve the sample extract with water and to remove the stickiness of the extracts. A commercial drug, glibenclamide, 10 mg/kg bw (Dibenol™), was used as a positive control for comparison with plant extracts. A measured amount of glibenclamide powder and a proper amount of distilled water was mixed to make a suspension of glibenclamide.

#### Experimental design

Alloxan-induced diabetic rats were assembled randomly into six groups denoted as Group 1: no treatment, normal food, and water; Group 2: alloxan-induced rats treated with glibenclamide, 10 mg/kg bw; Group 3: alloxan-induced rats with no other treatment; Group 4: alloxan-induced rats treated with methanol extract of leaves (MEL), 300 mg/kg bw; Group 5: alloxan-induced rats treated with methanol extract of bark (MEB), 300 mg/kg bw; Group 6: alloxan-induced rats treated with aqueous extract of leaves (AEL), 300 mg/kg bw. Each group included five Wistar albino rats. The experiment was carried out for 3 weeks (Khatun et al. 2015).

#### Estimation of glucose level

A glucometer was used for determining blood glucose concentrations of experimental animals. Blood glucose levels were assessed after overnight fasting (12–14 h) on days 0, 7, 14, and 21 after diabetes induction. The blood glucose was measured in mmol/L.

### Evaluation of antioxidant activity

Plant extracts were assessed for efficacy in scavenging free radicals using 1,1-diphenyl-2-picrylhydrazyl (DPPH) following Brand-Williams et al. (1995). Methanol solution (2.0 mL) of test specimens [plant extracts and control, i.e., butylated hydroxytoluene (BHT)] were mixed with 3.0 mL of a DPPH methanol solution (20 µg/mL). The range of concentrations of methanol solutions of test specimens was 500–0.977 µg/mL. A mixture of each of the methanol solutions of test specimens and DPPH solution was kept in the dark for 30 min at room temperature to complete the reaction and subsequently absorbance at 517 nm against methanol as blank was measured with a UV spectrophotometer. Inhibition percentage (I%) was calculated using the following formula:

$$\%\text{Inhibition}=\left(I-A_{\text{sample}}/A_{\text{blank}}\right)\times 100$$

Where *A*_blank_ is the absorbance of the control reaction (containing all the reagents except the test material) and *A*_sample_ is the absorbance of the sample.

The concentration of extracts that exhibited 50% inhibition (IC_50_) was computed by plotting inhibition percentage against extract concentrations.

### Evaluation of cytotoxicity

Methanol extracts of leaf and bark and the AEL of *S. asper* were assessed for cytotoxic properties according to Meyer et al. (1982). Brine shrimp eggs were hatched in simulated seawater for 48 h at room temperature. Test specimens were prepared by dissolving extracts at varying concentrations *viz*. 400, 200, 100, 50, 25, 12.5, 6.25, 3.12, 1.56, and 0.78 μg/mL in dimethyl sulfoxide (DMSO). Afterward, 10 brine shrimp larvae were incubated with the test specimens in triplicate vials. A mixture of seawater and DMSO was used as a control in this assay. All the vials were observed after 24 h using a lighted background to identify the number of surviving larvae in each vial.

### Statistical analysis

All values were expressed as mean ± SEM. Data were analyzed by one-way analysis of variance (ANOVA) followed by Student’s *t*-test.

## Results and discussion

### Antidiabetic effect

The present study demonstrated that extracts of leaf and bark of *S. asper* possessed notable antidiabetic activity. Recurring oral administration of the methanol extract of leaf and bark, and AEL (300 mg/kg) exhibited a significant reduction in the fasting blood glucose levels on different days after alloxan administration compared with normal and diabetic control groups rats (Table 1). After administration of MEB, blood glucose level was reduced from 19.82 to 5.88 mmol/L after 21 days of treatment. The positive standard, glibenclamide, reduced sugar level from 14.36 to 5.3 mmol/L after 21 days of treatment. The MEB showed a significant antidiabetic effect almost equal to glibenclamide (Table 1).

**Table 1.** Effect of different extracts of *Streblus asper* on blood glucose level in alloxan-induced diabetic rats.

Test models	Groups	Fasting blood glucose levels (mmol/L) Mean ± SEM			
		Day 0	Day 7	Day 14	Day 21
Normal	Group1. Normal control	5.66 ± 0.32	5.8 ± 0.31	5.7 ± 0.29	5.84 ± 0.13
Diabetic	Group 2. Glibenclamide	14.36 ± 0.93	6.2 ± 0.18	5.8 ± 0.12	5.3 ± 0.26
	Group 3. Diabetic control	13.14 ± 1.13	18.08 ± 1.26	23.28 ± 1.17	27.64 ± 0.45
	Group 4. MEL	19.06 ± 0.96	15.84 ± 1.25	14.08 ± 0.98	12.04 ± 0.64
	Group 5. MEB	19.82 ± 2.21	9.24 ± 1.59	6.7 ± 0.73	5.88 ± 0.52
	Group 6. AEL	14.38 ± 2.22	10.34 ± 1.99	8.24 ± 1.21	6.8 ± 0.57

MEL: methanol extract of leaf; MEB: methanol extract of bark; AEL: aqueous extract of leaf.

**Figure 1.**
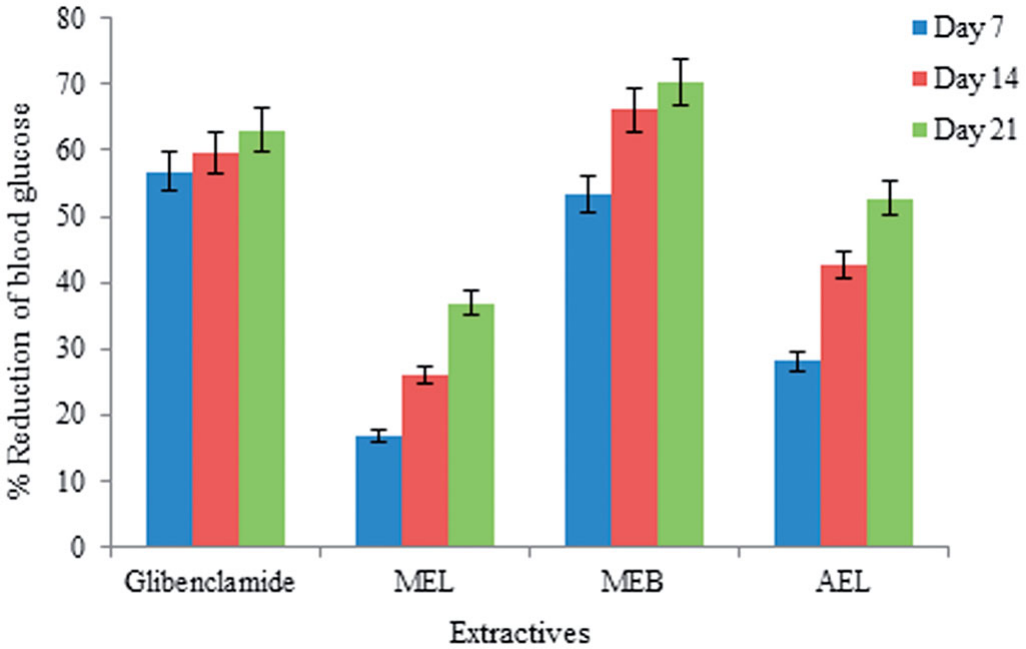
Percentage of reduction of blood glucose levels by different extracts of *Streblus asper* at different day interval. MEL: methanol extract of leaf; MEB: methanol extract of bark; AEL: aqueous extract of leaf.

Oral administration (once in a week which continued for consecutive 3 weeks) of methanol extract of leaves and bark, and AEL of *S. asper* to diabetic rats notably reduced blood glucose by 36.83, 70.33, and 52.71%, respectively, after 21 days of treatment. Glibenclamide showed a 63.09% reduction in blood glucose, while MEB reduced glucose by 70.33%, more than any other treatment. Percent reduction in blood glucose level for glibenclamide and different extracts is displayed in Figure 1.

The significance of methanol extracts of leaves and bark, and AEL of *Streblus asper* is depicted in Table 2.

**Table 2.** Significance of antidiabetic effect of different extracts of *Streblus asper*.

Test samples	*F* test value	*p*-Value	Level of significance
Glibenclamide (STD)	21.07	˂0.05	Statistically significance
MEL	4.09	˂0.05	Statistically significance
MEB	8.19	˂0.05	Statistically significance
AEL	20.84	˂0.05	Statistically significance

MEL: methanol extract of leaf; MEB: methanol extract of bark; AEL: aqueous extract of leaf.

In experimental animal models, induction of diabetes by Alloxan monohydrate is widely accepted (Carvalho et al. 2003). Generally, a single dose of 120 mg/kg bw of alloxan monohydrate is effective in inducing DM in rats, and we also found elevated glucose levels in the blood of fasting rats, collected from tails of the rats 48 h after administration. Usually, glucose transporter 2 plays a vital role in the accumulation and transportation of alloxan that leads to selective damage of pancreatic β-cells responsible for the production of insulin, and, thus, production of insulin is reduced. Consequently, the body suffers from insulin deficiency that affects glucose absorption by peripheral tissue resulting in diabetes. Moreover, alloxan acts as a catalyst and accelerates some redox reactions which produces free radicals that injure tissues and plays a pivotal role in dysregulation and degeneration of β-cells (Carvalho et al. 2003; Muhtadi et al. 2015).

It has been reported that the hyperglycemia-induced onset of diabetic complications is due to the impairment in the equilibrium between reactive oxygen species capacity and antioxidant defense capacity (Niedowicz and Daleke 2005; Misbah et al. 2013; Spadiene et al. 2014). Therefore, antioxidant agents can help scavenge various reactive oxygen species and prevention of diabetes mellitus. Methanol extracts of plants display a high proportion of phenolic compounds, saponins, tannins, alkaloids, carbohydrates, glycosides, and flavonoids (Murugan and Natarajan 2016). Methanol extracts in the current study are found to be effective for control of increased blood glucose levels, and this antihyperglycemic effect could be due to antioxidant phytochemicals, e.g., flavonoids, polyphenols, and tannins in the extract (Muhtadi et al. 2015). These antioxidants produce antidiabetic effects individually and/or participate in some sort of synergism. In agreement with our study, Kumar et al. (2012) demonstrated that methanol extract of *S. asper* root produced antidiabetic actions in STZ-induced diabetic rats and showed antioxidants present in the extract together exhibited hypoglycaemic effects. Alloxan induces degenerative changes in β-cells and causes strong morphological changes in diabetic rats with severe pancreatic β-cells destruction, such as decreasing the islets cell numbers, cell damage, and cell death (Al-Khalifa et al. 2009; Dahech et al. 2011). The cytotoxicity of alloxan is mediated by ROS (Reactive oxygen species), with a simultaneous massive increase in cytosolic calcium concentration leading to the rapid destruction of β-cells (Bahar et al. 2016; Bathina et al. 2016). The polyphenols and flavonoids available in plant extract are found to exert β-cell protective effects through reducing ROS production and decreasing cell apoptosis (Bahar et al. 2017). Other underlying mechanisms are also possible, e.g., increased rate of filtering might accelerate the removal of glucose from circulation, renal excretion, and increased discharge of glucose by unregulated metabolic processes or adsorption into the fat layer may also occur (Muhtadi et al. 2015).

### Antioxidant potential

**Figure 2.**
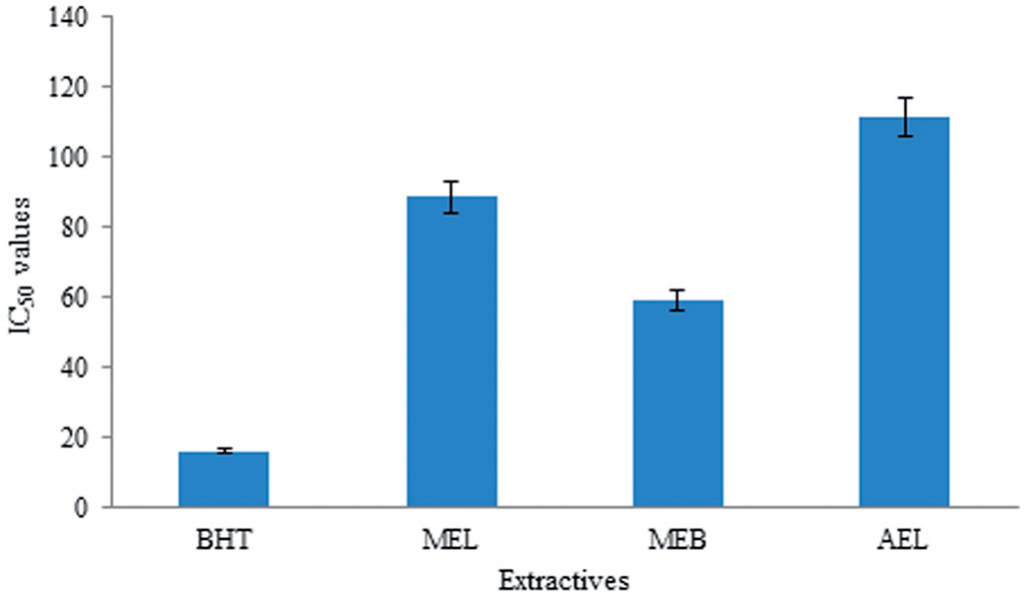
IC_50_ values of standard and different extracts of *Streblus asper*. BHT: *tert*-butyl-1-hydroxytoluene; MEL: methanol extract of leaf; MEB: methanol extract of bark; AEL: aqueous extract of leaf.

The abilities of different extracts and standard compounds to scavenge DPPH radicals are presented in Table 3, and the corresponding IC_50_ values are depicted in Figure 2. Lower IC_50_ reflects higher scavenging abilities. DPPH radical scavenging activities in the MEB > MEL > AEL (*p* < 0.05). The MEB of *S. asper* shows the strongest antioxidant potential with IC_50_ values of 58.9 µg/mL, which is moderate compared to BHT (IC_50_: 16.17 µg/mL) (Table 3; Figure 2).

**Table 3.** IC_50_ for DPPH radical scavenging properties of different extracts of *Streblus asper*.

Abs. of blank	Conc. (µg/mL)	Absorbance of the extracts				% of inhibition				IC_50_ (µg/mL)			
		BHT	MEL	MEB	AEL	BHT	MEL	MEB	AEL	BHT	MEL	MEB	AEL
0.325	500	0.02	0.10	0.10	0.14	95.07	67.69	68.92	64				
	250	0.02	0.13	0.11	0.15	94.15	60.92	65.53	58.15				
	125	0.03	0.15	0.14	0.15	89.54	55.08	57.84	55.38				
	62.5	0.07	0.19	0.15	0.17	80	42.74	54.46	46.77				
	31.25	0.11	0.19	0.19	0.19	65.53	39.08	41.84	44.61				
	15.63	0.18	0.23	0.22	0.21	44.31	29.23	32.00	35.38				
	7.81	0.22	0.24	0.24	0.22	31.70	27.38	26.80	31.08				
	3.91	0.27	0.24	0.24	0.22	16.92	25.23	25.85	28.62	16.17	88.54	58.92	111.36
	1.95	0.27	0.26	0.25	0.24	16.62	21.23	22.15	26.46				
	0.98	0.28	0.26	0.25	0.25	15.08	12.31	15.68	21.23				

BHT: *tert*-butyl-1-hydroxytoluene; MEL: methanol extract of leaf; MEB: methanol extract of bark; AEL: aqueous extract of leaf.

**Figure 3.**
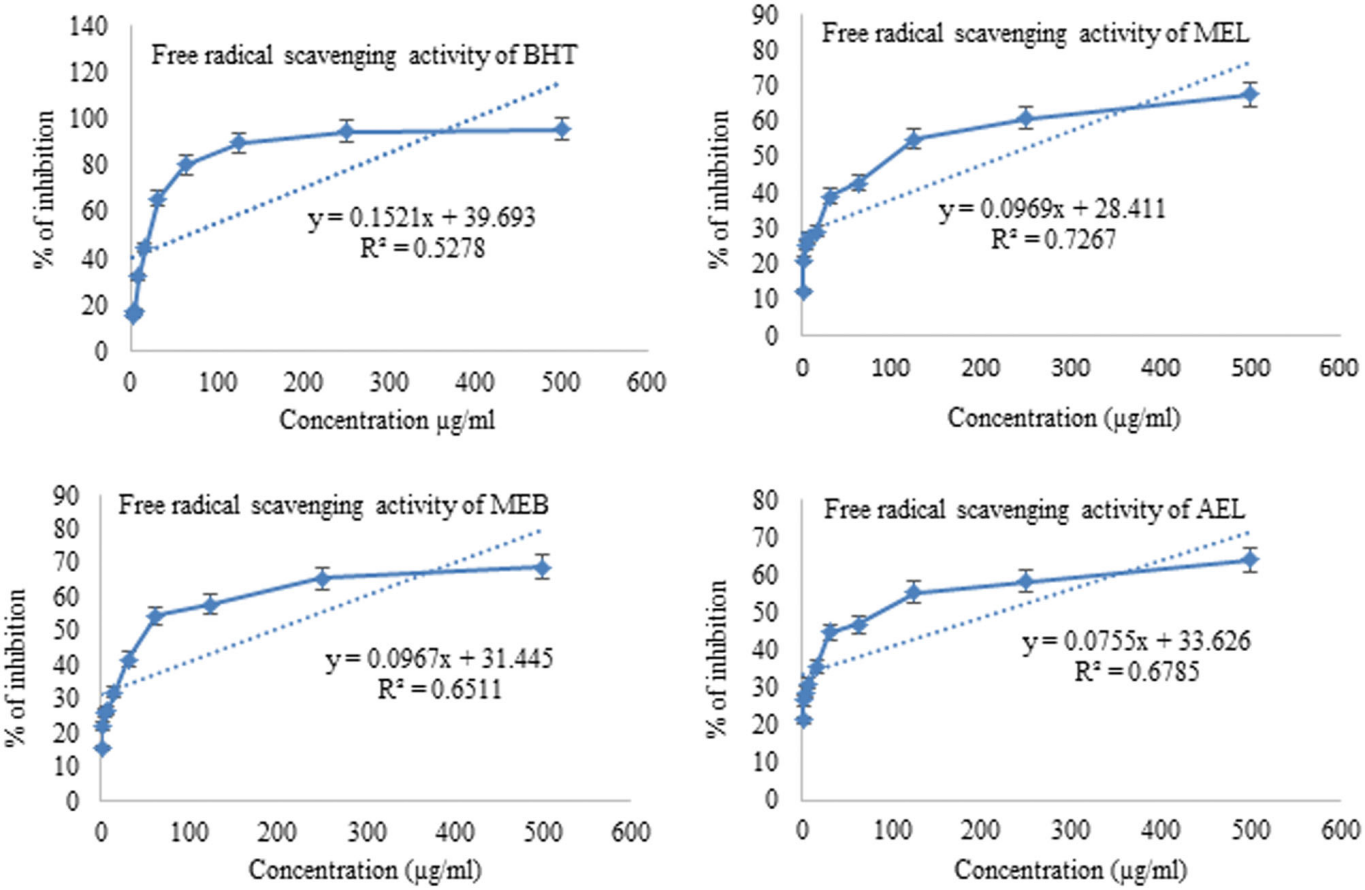
Plot of % inhibition and predicted regression line of different extracts of *Streblus asper*. BHT: *tert*-butyl-1-hydroxytoluene; MEL: methanol extract of leaf; MEB: methanol extract of bark; AEL: aqueous extract of leaf.

Dose-response curves of scavenging abilities of extracts show increases with increasing concentrations demonstrating dose-dependency (Figure 3). MEB showed 58.92% radical scavenging activity at a concentration of 500 µg/mL. This result is consistent with Kakoti et al. (2007) and Kumar et al. (2012), where methanol extract of different parts has been reported to possess moderate to high DPPH radical scavenging abilities. Phenolic compounds can show strong antioxidant properties, and a significant correlation is observed between the total phenolic content of extracts and the DPPH radical scavenging ability of extracts (Soobrattee et al. 2005). Kakoti et al. (2007) reported that extracts of *S. asper* are rich in phenolic content and observed antioxidant activities are primarily attributed to phenolic compounds.

Oxidative stress is reported to coexist with impairment in endogenous antioxidant status and removal of oxidative damage through activation of antioxidative enzymes, such as CAT, glutathione S-transferase (GST), glutathione peroxidase (GPx), etc. All chemical entities with antioxidant properties eliminate oxidative potential by inhibiting free radicals and lipid peroxidation and enhancing GSH and CAT activities (Das et al. 2011). Earlier studies demonstrate that alcoholic extracts of *S. asper* restore hepatic biomarkers, such as SGOT, SGPT, SALP, total cholesterol, and serum triglycerides, towards normal levels to maintain blood glucose within the normal range (Kumar et al. 2012). Treatment with alcoholic extracts increases depleted GSH and lessens CAT, thus reducing oxidative damage in the liver and kidney (Kumar et al. 2012). Therefore, DPPH free radical scavenging properties, i.e., the antioxidant properties of *S. asper*, might also be due to the presence of phenolic compounds which in turn, exhibit antihyperglycemic activities in alloxan-induced diabetic rats.

### Cytotoxicity

The percentage of shrimps that died after 24 h was plotted against the logarithm of concentrations on test solutions to determine lethal concentrations (LC_50_) and the best fit line was obtained by regression analysis. LC_50_ for vincristine sulphate (VS) was 2.48 µg/mL and LC_50_’s of MEL, MEB, and AEL were 173.80, 32.36, and 3235.9 µg/mL, respectively (Table 4). Effects of different extracts and the positive control, VS, at different concentrations on shrimp nauplii are shown in Table 5.

**Table 4.** LC_50_ for Brine Shrimp lethality of different extracts of *Streblus asper*.

Test samples	Regression line	*R* ^2^	LC_50_ (µg/mL)
VS	*y* = 25.77x + 39.854	0.9622	2.48
MEL	*y* = 21.898x + 0.8741	0.755	173.80
MEB	*y* = 31.624x + 2.7134	0.7982	32.36
AEL	*y* = 14.207x + 0.301	0.736	3235.9

VS: vincristine sulphate; MEL: methanol extract of leaf; MEB: methanol extract of bark; AEL: aqueous extract of leaf.

**Table 5.** Effect of different extracts of *Streblus asper* on shrimp nauplii in brine shrimp lethality test.

Conc. (µg/mL)	Log_10_ conc.	No. of nauplii taken	% of mortality				LC_50_ value (µg/mL)			
			VS	MEL	MEB	AEL	VS	MEL	MEB	AEL
00	–	–	–				4.28	173.80	32.36	3235.93
0.78125	−1.1072	10	20	0	0	0				
1.5625	0.18382	10	30	0	0	0				
3.125	0.49485	10	30	0	0	0				
6.25	0.79588	10	40	10	10	0				
12.5	1.09691	10	50	10	20	10				
25	1.39794	10	70	20	40	10				
50	1.69897	10	80	40	50	20				
100	2.0	10	80	40	80	30				
200	2.30103	10	100	60	90	40				
400	2.60206	10	2.47	80	100	50				

VS: vincristine sulphate; MEL: methanol extract of leaf; MEB: methanol extract of bark; AEL: aqueous extract of leaf.

**Figure 4.**
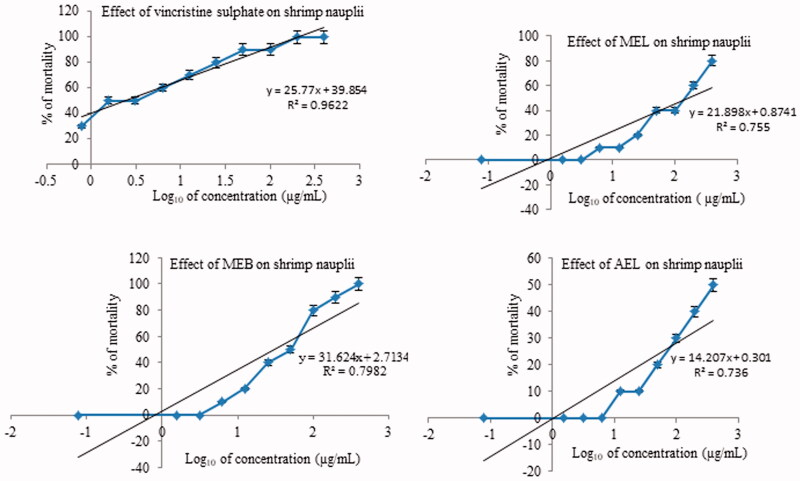
Plot of % mortality and predicted regression line of different extracts of *Streblus asper*. VS: vincristine sulphate; MEL: methanol extract of leaf; MEB: methanol extract of bark; AEL: aqueous extract of leaf.

Methanol extracts of leaf and bark showed dose-dependent cytotoxicity and percent mortality is found to increase with increasing concentrations of leaf and bark extracts (Figure 4).

The brine shrimp lethality bioassay model is a well-accepted and widely deployed model for the preliminary assessment of cytotoxic properties of plant extracts and pure compounds. In several studies, brine shrimp lethality assay results correlate with cytotoxic properties of extracts or compounds (Alluri et al. 2005). LC_50_ values for crude extracts <1 mg/mL are considered significantly active, implying that the AEL of *S. asper* with LC_50_ of 3235.9 µg/mL, is non-toxic. In contrast, in MEB and MEL, LC_50_’s of 32.36 and 173.78 µg/mL, respectively possess higher and moderate cytotoxicity. Generally, several reasons are encountered for herbal toxicity, e.g., variations in levels of active ingredients in different plant parts, harvesting time, stages of development, geography, and climatic conditions specific to the plant growth and development (Saad et al. 2006). Further, different chemical classes, such as terpenoids, steroids, saponins, lignins, and quinones are responsible for antitumor properties and cytotoxicity (Ripa et al. 2009), and methanol extracts contain a wide array of chemical classes. Our MEB of *S. asper* might contain all such chemical entities responsible for cytotoxicity that could work individually or in concert for ovicidal or larvicidal properties (Ripa et al. 2009). Consequently, the present results indicate that MEB should be further investigated to identify responsible compounds and uncover the underlying mechanism of cytotoxicity.

## Conclusions

In the present study, we examined different extracts of the traditional medicinal plant, *Streblus asper,* and their ability to address two global health concerns, i.e., diabetes mellitus and the need for antioxidants with fewer adverse effects. The methanol bark extract of *S. asper* exhibited marked antihyperglycemic effects on diabetes-induced Wistar albino rats and more importantly, prominent antioxidant and cytotoxic effects. Our results help provide a scientific rationale for the ethnomedicinal use of *S. asper* in Bangladesh for treating various ailments and establish a new window for further exploring this plant as a source of bioactive chemical entities in the agrochemical and pharmaceutical industries.
